# Smoking habits, attitudes and training among medical students of the Democritus University of Thrace

**DOI:** 10.1186/1617-9625-12-S1-A4

**Published:** 2014-06-06

**Authors:** Georgios-Marios Pantsidis, Dimitra-Iosifina Papageorgiou, Demosthenes Bouros

**Affiliations:** 1 Faculty of Medicine, Democritus University of Thrace, Alexandroupolis, 68100, Greece; 2 Faculty of Medicine, University of Thessaly, Larisa, 41110, Greece

## Background

Tobacco use continues to be the leading global cause of preventable death, contributing to the death of nearly 6 million people each year. Medical students, who are future doctors, have an important role to play in smoking cessation and prevention. The objective of this study was to estimate the prevalence of tobacco use among medical students of Democritus University of Thrace Medical School, and to evaluate their smoking-related attitudes and their training in tobacco issues they receive during their studies in University.

## Materials and methods

This study was conducted from March to May 2011. The students were asked to complete a modified version of the Global Professional Students' Survey (GHPSS) questionnaire. The final study population consisted of 233 randomly selected students in the 1st-6th year of medical studies.

## Results

Of the 233 students invited to participate, 229 submitted adequately completed questionnaires. Of this sample, 24% were smokers, 38.2% of whom had experimented with smoking at the age of 11-15 years. The banning of smoking in all enclosed public places was considered useful by 88.6% with a statistically significant difference between smokers and non-smokers (65.5% vs. 96%, p<0.001). Of the participants, 31% believed that slim/light and hand-rolled cigarettes are less harmful and only 8.1% had been taught cessation techniques and 17.8% the reasons why people smoke.

## Conclusions

The study shows that the prevalence of smoking among medical students in northern Greece is high compared with other countries. It is evident that the issue of tobacco use is not covered adequately and systematically by the Medical School curriculum.

